# Granisetron *vs* ondansetron: is it a question of duration of 5-HT_3_ receptor blockade?

**DOI:** 10.1038/sj.bjc.6600313

**Published:** 2002-05-03

**Authors:** P Blower, M Aapro

**Affiliations:** Poole House, Great Yeldham, Halstead, Essex CO9 4HP, UK; Clinique de Genolier, 1272, Genolier, Switzerland

## Abstract

*British Journal of Cancer* (2002) **86**, 1662–1663. DOI: 10.1038/sj/bjc/6600313 www.bjcancer.com

© 2002 Cancer Research UK

## Sir

We read with interest the publication by de Wit *et al* (2001) that there may be effective cross-over to granisetron after failure on ondansetron; both are selective 5-HT_3_ receptor antagonists, a class of drugs considered the ‘gold-standard’ antiemetic agents for the control of emesis in cancer patients undergoing chemo- and radiotherapy. While we support the outcome that granisetron demonstrates superior efficacy to ondansetron, an effect that has been previously reported by Terrey and Aapro (1996), we have some concerns about the study design and the authors' interpretation of the results.

Firstly, from the outset this study favours granisetron. We recognise that the trial of de Wit *et al* (2001) involved only a small number of patients (*n*=40), however, for the study to be a balanced cross-over design, patients should have been selected from a background of failure on both ondansetron and granisetron with previous chemotherapy cycles. Moreover, the authors fail to discuss the possibility of a psychological bias toward the introduction of the ‘new’ drug. Psychological factors can influence treatment success and are known to be associated with anticipatory nausea and vomiting, a negative phenomenon that can have potentially disabling effects on patients undergoing successive chemotherapy cycles. Conversely, there may be positive psychological elements benefiting the patient – the design of this trial does not control for this effect. In double-blind trials in which patients fail on one antiemetic treatment and then go on to receive the antiemetic in subsequent cycles, anticipation of a switch in therapy may be enough to demonstrate an improved response.

Despite its importance to the interpretation of the study results, de Wit *et al* (2001) have made no reference to the longer duration of action of granisetron *vs* ondansetron, nor the pharmacodynamic properties of the two agents. Compelling data accumulated from cutaneous flare experiments in human volunteers demonstrate that, following a single infusion of granisetron, 40 μg kg^−1^, the 5-HT_3_ receptor-mediated axon flare reflex is significantly reduced for at least 24 h (Cooper *et al*, 1988; Upward *et al*, 1990). This contrasts with a 2.6-fold shorter duration of action after infusion with ondansetron, 8 and 16 mg (Sweetland *et al*, 1992) and is further supported by the longer plasma half-life of granisetron (about 10 h; Carmichael *et al*, 1989) compared with ondansetron (about 3.8 h; Roila and Del Favero, 1995). Furthermore, granisetron differs from the competitive antagonist ondansetron in that it displays insurmountable antagonism at vagal afferent 5-HT_3_ receptors (Newberry *et al*, 1993), and it is this property that is thought to underlie the phenomenon that its pharmacodynamic half-life far exceeds its plasma half-life.

The authors fail to acknowledge further important data in their discussion. The two chemotherapeutic agents administered in this study, cisplatin (>=50 mg m^−2^) and cyclophosphamide (>=500 mg m^−2^), differ in their emetogenic potential as well as the time of onset of their emetic action (Doherty, 1999) (Table 1

**Table 1:**
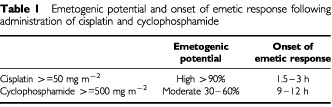
Emetogenic potential and onset of emetic response following administration of cisplatin and cyclophosphamide

). While de Wit *et al* (2001) report complete protection following cisplatin-based chemotherapy in two out of seven patients receiving granisetron and none out of six patients receiving ondansetron, the principal difference between the efficacy of ondansetron and granisetron in this study was following cyclophosphamide-based therapy. Fifty-eight per cent of patients receiving cyclophosphamide experienced complete protection of emesis following a single dose of granisetron, compared with 7% following a single dose of ondansetron; the later onset of emesis following cyclophosphamide must favour the longer duration of antiemetic effect of granisetron.

Additionally, de Wit *et al* (2001) state that, ‘there are no data to support the use of higher doses of the same 5-HT_3_ receptor antagonists in patients failing the recommended dosage,’ citing Gandara *et al* (1998). However, a dose-related response (*P*<0.001) for oral dolasetron has been shown between doses of 25 and 200 mg (complete response rates 60.5 and 76.3% for 100 and 200 mg dolasetron, respectively; Fauser *et al*, 1996), despite a recommended dosage for the agent of 100 mg. Similar dose-related effects have also been reported with ondansetron. For example, a Phase I study comparing three doses of intravenous ondansetron t.d.s. (0.015, 0.15 and 0.3 mg kg^−1^) indicated a trend towards increased efficacy with higher doses (complete response rates were 15, 46 and 58% respectively), though only the 0.015 mg kg^−1^ dose was shown to be significantly different from the other dose levels (Grunberg, 1992). Further studies have reported significantly superior efficacy with a single dose on ondansetron, 32 mg, compared with a single 8 mg dose (Beck *et al*, 1992; Tsavaris *et al*, 2001). Moreover, studies comparing granisetron, 40 and 160 μg kg^−1^, show a trend towards increased efficacy at the higher dose (Smith, 1993; Soukop, 1994); 24 h after administration of chemotherapeutic agents, 81% of granisetron, 160 mcg kg^−1^, were emesis-free compared with 75% receiving granisetron 40 μg kg^−1^ (Smith, 1993). In addition, patients refractory to other 5-HT_3_ receptor antagonists have been shown to respond well to granisetron, at doses that are higher than generally used, in subsequent chemotherapy cycles (Carmichael *et al*, 1998).

In conclusion, the effective ‘switch’ to granisetron in this study may, therefore, be due to a number of factors, but is likely to be attributable primarily to the longer duration of action of granisetron compared with ondansetron rather than the lack of cross resistance between 5-HT_3_ receptor antagonists.
